# Nrdp1 Increases Ischemia Induced Primary Rat Cerebral Cortical Neurons and Pheochromocytoma Cells Apoptosis Via Downregulation of HIF-1α Protein

**DOI:** 10.3389/fncel.2017.00293

**Published:** 2017-09-20

**Authors:** Yuan Zhang, Ke Yang, Ting Wang, Weiping Li, Xinchun Jin, Wenlan Liu

**Affiliations:** ^1^The Central Laboratory, Shenzhen Second People’s Hospital, Graduate School of Guangzhou Medical University Shenzhen, China; ^2^Shenzhen Key Laboratory of Neurosurgery, Shenzhen Second People’s Hospital, Graduate School of Guangzhou Medical University Shenzhen, China; ^3^Department of Pathophysiology, Baotou Medical College Baotou, China; ^4^Department of Neurosurgery, The First Affiliated Hospital of Shenzhen University, Shenzhen Second People’s Hospital Shenzhen, China; ^5^Jiangsu Key Laboratory of Translational Research and Therapy for Neuro-Psycho-Diseases and Institute of Neuroscience, The Second Affiliated Hospital of Soochow University, Soochow University Suzhou, China; ^6^School of Pharmacy, Key Laboratory of Molecular Pharmacology and Drug Evaluation, Ministry of Education, Yantai University Yantai, China

**Keywords:** ischemic stroke, neuronal injury, Nrdp1, apoptosis, HIF-1α, USP8

## Abstract

Neuregulin receptor degradation protein-1 (Nrdp1) is an E3 ubiquitin ligase that targets proteins for degradation and regulates cell growth, apoptosis and oxidative stress in various cell types. We have previously shown that Nrdp1 is implicated in ischemic cardiomyocyte death. In this study, we investigated the change of Nrdp1 expression in ischemic neurons and its role in ischemic neuronal injury. Primary rat cerebral cortical neurons and pheochromocytoma (PC12) cells were infected with adenoviral constructs expressing Nrdp1 gene or its siRNA before exposing to oxygen-glucose deprivation (OGD) treatment. Our data showed that Nrdp1 was upregulated in ischemic brain tissue 3 h after middle cerebral artery occlusion (MCAO) and in OGD-treated neurons. Of note, Nrdp1 overexpression by Ad-Nrdp1 enhanced OGD-induced neuron apoptosis, while knockdown of Nrdp1 with siRNA attenuated this effect, implicating a role of Nrdp1 in ischemic neuron injury. Moreover, Nrdp1 upregulation is accompanied by increased protein ubiquitylation and decreased protein levels of ubiquitin-specific protease 8 (USP8) in OGD-treated neurons, which led to a suppressed interaction between USP8 and HIF-1α and subsequently a reduction in HIF-1α protein accumulation in neurons under OGD conditions. In conclusion, our data support an important role of Nrdp1 upregulation in ischemic neuronal death, and suppressing the interaction between USP8 and HIF-1α and consequently the hypoxic adaptive response of neurons may account for this detrimental effect.

## Introduction

Cerebral ischemia initiates a cascade of cytotoxic molecules responsible for the death of neural cells as well as the damage of the blood brain barrier (BBB) at the injury site (Doyle et al., 2008). In more than one decade, ischemia-associated neuronal injury has been a topic of intensive investigation, which leads to the identification of several mechanisms accounting for cerebral ischemia injury, such as apoptosis, oxidative damage, inflammatory injury, mitochondrial dysfunction and dysregulated protein degradation (Caldeira et al., 2014; Kalogeris et al., 2014; Palencia et al., 2015). The ubiquitin-proteasome system (UPS) is the major intracellular machinery for protein degradation, which is responsible for maintaining cellular homeostasis by regulating several important processes such as cell death, cell division, cell signal transduction, cell cycle progression and transmembrane transport (Wagner et al., 2011). Emerging evidence has suggested a role of suppressed proteasome activity in contributing to neuronal death in ischemic brain injury. However, little is known about the UPS components whose activities are suppressed under brain ischemia conditions (Caldeira et al., 2014).

Neuregulin receptor degradation protein-1 (Nrdp1, also known as FLRF or RNF41), is a ring finger E3 ubiquitin ligase and primarily expressed in the brain, heart, prostate and skeletal muscle (Diamonti et al., 2002). Several studies have demonstrated an important role of Nrdp1 in regulating cell growth, apoptosis, oxidative stress and inflammation, in which Nrdp1 promotes the ubiquitination of ubiquitin-specific protease 8 (USP8), ErbB3, ErbB4, BRUCE/Apollon, MyD88 and Parkin (Qiu et al., 2004; Yen et al., 2006; Yu and Zhou, 2008; Wang et al., 2009; De Ceuninck et al., 2013; Sun et al., 2017). In a previous study, we have shown that Nrdp1 is implicated in ischemic cardiomyocyte death, in which overexpression of Nrdp1 augmented ischemia–reperfusion (I/R)-induced cardiomyocyte apoptosis while inhibition of endogenous Nrdp1 could protect cardiomyocytes against I/R injury (Zhang et al., 2011b). In the brain, Nrdp1 is found to be involved in suppressing brain tumor formation and promoting lipopolysaccharide (LPS)-induced neuroinflammation via its pro-apoptotic action (Shen et al., 2015; Shi et al., 2015; Wu et al., 2016). It is well known that neuronal apoptosis is a major event in ischemic stroke, however, the role of Nrdp1 in ischemic neuronal death has not yet been investigated. Our preliminary data shows that cerebral ischemia induces Nrdp1 mRNA expression in ischemic cerebral cortex in a rat model of middle cerebral artery occlusion (MCAO). However, the exact role of Nrdp1 in ischemia-induced neuronal damage remains to be determined.

USP8 is a substrate of Nrdp1, and interestingly, it is also a de-ubiquitination enzyme (Wu et al., 2004; De Ceuninck et al., 2013). This de-ubiquitination activity has made USP8 a stabilizing molecule for HIF-1α protein, in which USP8 prevents HIF-1α from pVHL-mediated degradation (Troilo et al., 2014). It is well known that HIF-1α plays a vital role in attenuating brain tissue damage through promoting adaptive response during ischemic stroke (Helton et al., 2005; Baranova et al., 2007; Fan et al., 2009; Singh et al., 2012; Zhang et al., 2014; Yang Y. et al., 2017). These data raise an important hypothesis that ischemia-induced Nrdp1 upregulation may contribute to ischemic neuronal injury via downregulating USP8 (via degradation) and thus destabilizing HIF-1α in ischemic neurons.

In the present study, we tested this hypothesis in cultured primary cerebral cortical neurons and PC12 cells (pheochromocytoma of the rat adrenal medulla) using an *in vitro* ischemic model of oxygen-glucose deprivation (OGD). We chose relative short OGD durations as the ischemic stimulus in this study because we have been focusing on early ischemic BBB damage that occurs within the first 4.5 h after ischemia onset (i.e., the therapeutic time window of tPA thrombolysis for ischemic stroke; Hacke et al., 2008; Jin et al., 2012; Liu et al., 2012, 2016), and in these studies, we observed substantial neuronal death in the ischemic brain within several hours after stroke onset (Jin et al., 2012; Liu et al., 2012). Our data showed that OGD treatment significantly increased Nrdp1 expression in neuronal cells, and knockdown or overexpression of Nrdp1 augmented or attenuated OGD-induced neuronal death, respectively. Moreover, Nrdp1 upregulation was accompanied by increased ubiquitinization of USP8 and its degradation, and this change was associated with decreased HIF-1α levels in ischemic neurons.

## Materials and Methods

### Rat Model of MCAO

The Laboratory Animal Care and Use Committee of Shenzhen University approved all animal related experimental protocols. Male Sprague–Dawley rats (purchased from the Experimental Animal Center Southern Medical University, Guangzhou, Guangdong, China) weighing 300 g to 400 g were anesthetized with isoflurane (4% for induction, 1.75% for maintenance) in N_2_O:O_2_ (70%:30%) during surgical procedures and the body temperature was maintained through a heated pad. A 4–0 silicone-coated monofilament nylon suture was introduced into the right intra-carotid artery to occlude the opening of the MCA as we previously described (Liu et al., 2009). MCAO was lasted for 3 h, and the animals were then deeply anesthetized with isoflurane and euthanized by decapitation. Successful MCAO was confirmed by 2,3,5-triphenyltetrazolium chloride (TTC, Sigma-Aldrich, St. Louis, MI, USA) staining of the 2-mm-thick brain coronal section 6 mm away from the tip of the front lobe as we previously described (Liu et al., 2008).

### Primary Culture of Rat Cerebral Cortical Neurons

Rat primary cortical neurons were cultured using a method as we described previously (Liu et al., 2007). Briefly, cerebral cortices were removed from the embryos of Sprague–Dawley pregnant rats at 15–18 days gestation (Shouthern Medical University Experimental Animal Center). After removing the meninges, the cortical tissue was minced and incubated with 0.05% trypsin for 30 min at 37°C with gentle trituration. After digestion, the neurons were achieved and suspended in neurobasal medium containing 2% B27 supplement and 0.5 mM L-Glutamine. Before seeding, culture vessels including 96-well plates, 1.2 cm glass slides or 6 cm dishes were coated with poly-L-lysine (PLL; 50 μg/mL, Sigma-Aldrich) at room temperature overnight. Neurons were maintained at 37°C in a humidified 5% CO_2_ incubator and half of the culture medium was changed every 3 days. The neurons were subjected to experiments 8 days after seeding.

### PC12 Cells Culture

PC12 cells, a rat PC12 cell line, were obtained from the Cell Resource Center of the Institute of Basic Medical Sciences, Peking Union Medical College/Chinese Academy of Medical Sciences (Beijing, China). PC12 cells were grown as a monolayer in dulbecco’s modified eagle medium (DMEM) supplemented with 10% horse serum and 5% fetal bovine serum (FBS), 100 U/ml penicillin and 100 μg/ml streptomycin at 37°C in a humidified incubator gassed with 5% CO_2_ and 95% room air. For neuronal differentiation, PC12 cells were seeded in PLL pre-coated plates and allowed to adhere for 24 h. Following adherence, the culture medium was replaced with nerve growth factor (NGF, 50 ng/ml; New England Biolabs, MA, USA) containing medium. The NGF-containing medium was replaced every other day. Seven days after the supplement of NGF, the NGF-induced differentiation of PC12 cells were determined using immunofluorescence staining with antibodies against the neuron-specific marker microtubule associated protein 2 (MAP2; Supplementary Figure S1).

### Adenoviral Constructs and Transfection

Recombinant adenoviral constructs, including Ad-control (control construct), Ad-Nrdp1 (overexpressing Nrdp1), Ad-si-control (expressing non-targeting control siRNA) and Ad-si-Nrdp1 (expressing Nrdp1 siRNA) were generated as described previously (Zhang et al., 2011a). Eight days after plating, cells were infected with Ad-control, Ad-Nrdp1, Ad-si-control or Ad-si-Nrdp1 for 24 h before OGD treatment.

### OGD Treatment

To mimic ischemic condition *in vitro*, primary neurons or PC12 cells were exposed to OGD as described previously (Liu et al., 2012). In brief, neurons or PC12 cells were subjected to OGD by replacing the normal growth medium with glucose free medium (DMEM without glucose) pre-equilibrated with 95% N_2_ and 5% CO_2_. The cells were then incubated in a humidified airtight chamber (Biospherix Ltd., Lacona, NY, USA) for 1 h, 3 h, or 6 h. Control cultures were incubated with normal DMEM medium without FBS at 37°C in 5% CO_2_/95% air. OGD was terminated by removing cells from the hypoxic chamber and the cells were collected separately for further measurement.

### Lactate Dehydrogenase (LDH) Release Assay

After OGD treatment, cell cytotoxicity was determined by the release of lactate dehydrogenase (LDH), a cytoplasmic enzyme released from cells. LDH release into the culture medium was detected using a CytoTox 96^®^Non-Radioactive Cytotoxicity Assay Kit (Promega Corporation. Madison, WI, USA). Briefly, 50 μl of each sample medium (i.e., pure culture medium for measuring background LDH release, culture media collected from control or OGD-treated cells for measuring experimental LDH release and lysis buffer-treated cells for measuring maximum LDH release) was collected to assay LDH release. The samples were incubated with reduced form of nicotinamide adenine dinucleotide and pyruvate for 30 min at room temperature and the reaction was terminated by adding Stop Solution. LDH release was assessed by measuring the absorbance of supernatants at 490 nm. Cell death rate was calculated as follows: cell death rate = (experimental LDH release-background LDH release) /(maximum LDH release-background LDH release) × 100%. The results were presented as fold increase of the control cells.

### Real-Time RT-PCR

Total RNA was isolated from neurons using Trizol reagents (Invitrogen Life Technologies, Carlsbad, CA, USA). RNA samples (2 μg) were reverse-transcribed to generate first-strand cDNA. After reverse transcription using TaqMan^®^ Reverse Transcription Kits (Applied Biosystems), reverse-transcribed products were amplified with the 7900HT real-time PCR System (Applied Biosystems) using SYBR^®^ Green PCR Master Mix (Applied Biosystems, Foster City, CA, USA) under the following conditions: 30 s at 95°C, followed by a total of 40 cycles of two temperature cycles (15 s at 95°C and 1 min at 60°C). Primer sequences were as follows: rat Nrdp1 forward: 5′-ATGGGGTATGATGTAACCCGG-3′ and reverse: 5′-GATGCAGGCGTTGCAGAAG-3′; Rat GAPDH served as endogenous control, and the primers were forward: 5′-CAATGTGTCCGTCGTGGATCT-3′; reverse: 5′-GTCCTCAGTGTAGCCCAAGATG-3′. The Ct value was calculated by the comparative ∆∆C_t_ method using the SDS Enterprise Database software (Applied Biosystems).

### Determination of Cell Apoptosis Rate

Apoptosis was analyzed by TUNEL assay using Click-iT^®^ Plus TUNEL Assay (Life Technologies, Inc., Carlsbad, CA, USA) according to manufacturer’s instruction. Briefly, at the end of the indicated treatments, primary rat cerebral cortical neurons grown on coverslips were incubated with TdT reaction mixture for 2 h at 37°C, followed by 30-min incubation with the Alexa Fluor^®^ 594 dye. Then, the cells were counterstained with DAPI (Sigma-Aldrich) for 20 min and observed under a fluorescence microscope (Leica, Germany). The TUNEL-positive nuclei of six non-overlapping fields per coverslip were counted by a researcher blinded to treatment, and these counts were converted to percentages by comparing the TUNEL-positive counts to the total number of cell nuclei as determined by DAPI counterstaining, that is TUNEL-positive ratio = (number of red nuclei/number of blue nuclei) × 100%.

### Western Blot Analysis

Protein samples were prepared from cultured neurons or PC12 cells using extraction buffer as described previously (Liu et al., 2012). The protein samples were electrophoresed on SDS-PAGE gels and transferred to a nitrocellulose membrane. After blocking with 5% non-fat milk for 1 h at room temperature, the membranes were incubated with the indicated primary antibodies overnight and then with horseradish peroxidase-conjugated secondary antibody for 1 h. The blots were developed using a chemiluminescent system, and the bands were scanned, and densitometry analysis was performed with Gel-pro 4.5 Analyzer (Media Cybernetics, Silver Spring, MD, USA). The primary antibodies were anti-cleaved-PARP, anti-PARP, anti-Bcl-2, anti-Bax, anti-USP8 and anti-β-actin. Anti-Nrdp1 and anti-HIF-1α antibodies were purchased from BETHYL Laboratories and Novus Biologicals, respectively, and the rest primary antibodies were purchased from Santa Cruz Biotech. Relative protein levels were quantified after normalization to the loading control β-actin.

### Co-Immumoprecipitation

After the indicated treatments, PC12 cells were lysed in RIPA buffer and centrifuged at 12,000× *g* for 10 min at 4°C. The supernatant were incubated overnight at 4°C with 4 μg of anti-USP8 (Santa Cruz Biotech, Chicago, IL, USA), followed by precipitation with 50 μl of Dynabeads protein A (Pierce Biotechnology, Rockford, IL, USA) for 10 min at room temperature. The protein A were then washed extensively with binding buffer, resuspended in SDS-PAGE buffer, and boiled for 5 min. Samples of 30 μg total cell lysate were used as an input control. The precipitated complexes were separated on SDS-PAGE gels, and transferred to nitrocellulose membranes, and immunoblotted with anti-Nrdp1 (BETHYL Laboratories, Montgomery, TX, USA), K48-linkage-specific anti-ubiquitin antibody (Abcam, Cambridge, MA, USA) or anti-HIF-1α antibody (Novus Biological, Littleton, CO, USA) to detect the presence of these proteins in the complex. Normal rabbit IgG (Santa Cruz Biotech) was used as a loading control.

### Statistical Analysis

All data were expressed as means ± SEM. Differences between groups were evaluated by either an unpaired Student’s *t* test or one-way ANOVA followed by Tukey’s *post hoc* test as indicated in the Figure Legends. *P* < 0.05 was regarded as statistically significant.

## Results

### Nrdp1 Is Upregulated in Ischemic Cerebral Cortex in a Rat Model of Middle Cerebral Artery Occlusion

**Figure 1:**
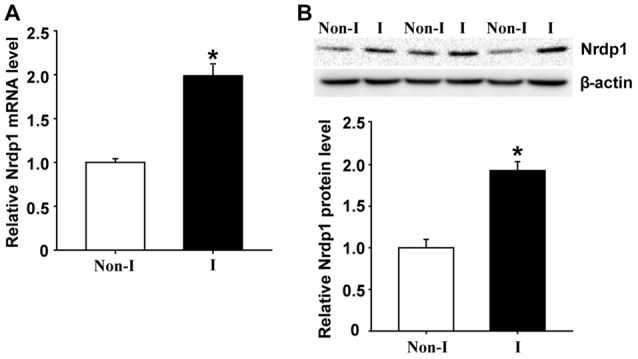
Middle cerebral artery occlusion (MCAO) induces neuregulin receptor degradation protein-1 (Nrdp1) upregulation in cerebral cortex. Rats were subjected to 3-h MCAO before isolating hemispheric cerebral cortex. The mRNA and protein levels of Nrdp1 in cerebral cortex from nonischemic (Non-I) and ischemic (I) hemispheric tissue were analyzed by real-time RT-PCR and western blot. **(A)** Real-time RT-PCR analysis showed that Nrdp1 mRNA expression was significantly increased in ischemic hemispheric cortex. *P < 0.05 vs. Non-I, ANOVA; *n* = 6. **(B)** Western blot analysis revealed increased levels of Nrdp1 protein in ischemic hemispheric cortex. Upper panel: representative immunoblots of Nrdp1 and the loading control β-actin; bottom panel: quantitative data of protein band intensity after normalization to β-actin. *P < 0.05 vs. Non-I, ANOVA; *n* = 6.

Nrdp1 is found to be widely expressed in the brain and is implicated in ischemic damage to the heart (Zhang et al., 2011b). To determine whether Nrdp1 plays a role in ischemic brain injury, we examined the change of Nrdp1 expression in the cerebral cortex isolated from the rats that were subjected to 3-h MCAO without reperfusion. Nrdp1 mRNA expression was analyzed in isolated hemispheric cortex by real time RT-PCR and found that 3-h MCAO induced a significant increase (~1-fold) of Nrdp1 mRNA expression in ischemic hemispheric cortex compared to non-ischemic cortical tissue (Figure 1A, *P* < 0.05). Consistent with its mRNA change, Nrdp1 protein levels were also significantly increased in ischemic cerebral cortex (Figure 1B). These results demonstrate that Nrdp1 is upregulated in the ischemic brain cortex. To further demonstrate a role of Nrdp1 in ischemic neuron injury and the underlying mechanisms involved, we chose the widely used *in vitro* model of ischemia (i.e., OGD) for the rest of this study.

### OGD Induces Nrdp1 Expression in Cerebral Cortical Neurons and PC12 Cells

**Figure 2:**
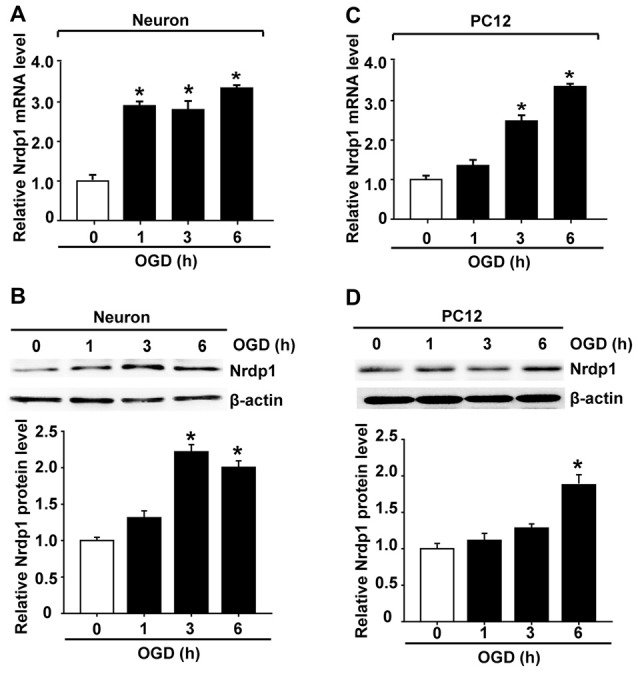
Oxygen-glucose deprivation (OGD) induces Nrdp1 upregulation in primary rat cerebral cortical neurons and PC12 cells. Cells were subjected to OGD treatment or normoxia (Control, Con) for 1, 3, or 6 h before analyzing Nrdp1 mRNA and protein expression using real-time RT-PCR and western blot. **(A)** Real time RT-PCR analysis showed that Nrdp1 mRNA expression was significantly increased in primary rat cerebral cortical neurons at 1 h after OGD treatment and was further increased when OGD was prolonged to 6 h. *P < 0.05 vs. Con, ANOVA; *n* = 4. **(B)** Western blot analysis showed that Nrdp1 protein levels were increased in 3-h OGD- and 6-h OGD-treated primary rat cerebral cortical neurons, but not in 1-h OGD-treated cells. Upper panel: representative immunoblots of Nrdp1 and the loading control β-actin; bottom panel: quantitative data of protein band intensity after normalization to β-actin. *P < 0.05 vs. Con, ANOVA; *n* = 4. **(C)** Real time RT-PCR analysis showed that Nrdp1 mRNA expression was significantly increased in PC12 cells at 3 h and 6 h after OGD treatment, but not 1 h. *P < 0.05 vs. Con, ANOVA; *n* = 4. **(D)** Western blot analysis showed that Nrdp1 protein levels were increased in 6-h OGD-treated PC12 cells, but not in 1-h OGD- and 3-h OGD-treated cells. Upper panel: representative immunoblots of Nrdp1 and the loading control β-actin; bottom panel: quantitative data of protein band intensity after normalization to β-actin. *P < 0.05 vs. Con, ANOVA; *n* = 4.

To determine the functional role of cerebral Nrdp1 in response to OGD treatment, we examined the expression of Nrdp1 in OGD-treated primary rat cerebral cortical neurons. The cells were exposed to OGD for 1, 3, or 6 h before analyzing Nrdp1 mRNA and protein levels. Real time RT-PCR analysis showed that Nrdp1 mRNA expression was increased in cerebral cortical neurons after exposing to OGD for 1 h and was further increased at 6-h OGD, while no significant difference was seen between 1-h OGD and 3-h OGD (Figure 2A). Western blot analysis showed that Nrdp1 protein levels were significantly increased in cerebral cortical neurons after exposing to OGD for 3 h and 6 h, but not for 1 h (Figure 2B). To further verify the above findings, we assayed the expression of Nrdp1 in PC12 cells exposed to the same OGD treatment as above. Real time RT-PCR analysis showed that Nrdp1 mRNA expression was increased in PC12 cells after exposing to OGD for 3-h and 6-h OGD, but not for 1 h (Figure 2C). Western blot analysis showed that Nrdp1 protein levels were significantly increased in PC12 cells after exposing to OGD for 6 h, but not for 1 h and 3 h (Figure 2D). These data demonstrate that OGD induces Nrdp1 upregulation in cerebral cortical neurons as well as PC12 cells in a time-dependent manner.

### Effects of Nrdp1 on OGD-Induced Apoptosis in Cerebral Cortical Neurons and PC12 Cells

**Figure 3:**
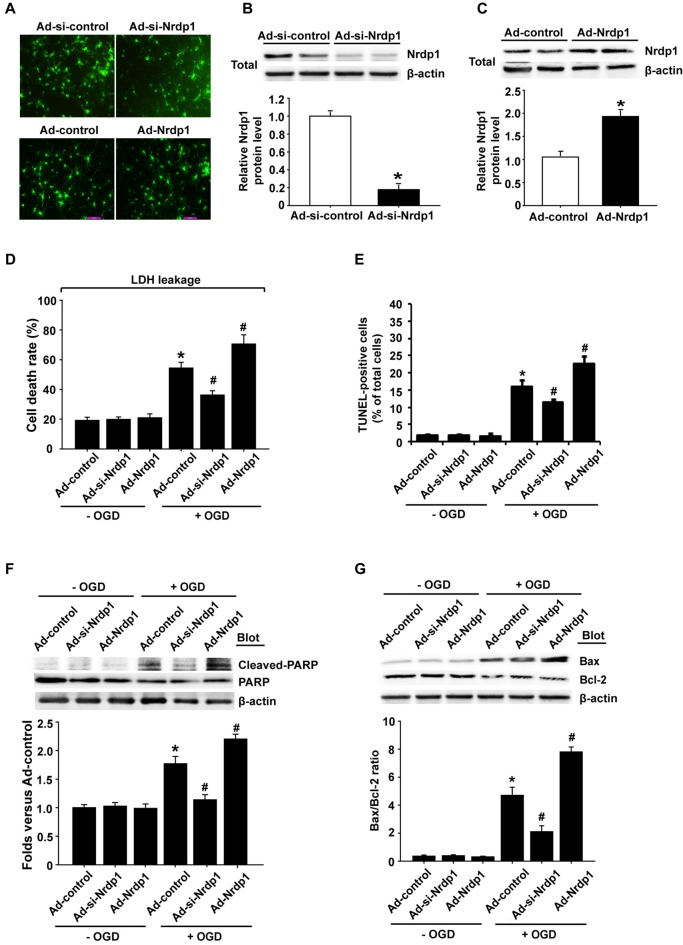
Effects of Nrdp1 on OGD-induced apoptosis in primary rat cerebral cortical neurons. **(A)** The infection efficiency of neurons with Ad-control, Ad-Nrdp1, Ad-si-control and Ad-si-Nrdp1 was visualized for green fluorescent protein (GFP) 24 h later using fluorescence microscopy (magnification, ×400). **(B)** Western blot analysis showed that incubation neurons with Ad-si-control and Ad-si-Nrdp1 for 48 h significantly (~90%) reduced Nrdp1 protein levels. Upper panel: representative immunoblots of Nrdp1 and the loading control β-actin; bottom panel: quantitative data of protein band intensity after normalization to β-actin. *P < 0.05 vs. Ad-si-control, ANOVA; *n* = 4. **(C)** Western blot analysis showed that incubation neurons with Ad-control and Ad-Nrdp1 for 48 h significantly increased Nrdp1 protein levels. Upper panel: representative immunoblots of Nrdp1 and the loading control β-actin; bottom panel: quantitative data of protein band intensity after normalization to β-actin. *P < 0.05 vs. Ad-control, ANOVA; *n* = 4. **(D)** Neurons were infected by with Ad-control, Ad-Nrdp1 or Ad-si-Nrdp1 and then treated with OGD for 6 h. Cell death rate was assessed by lactate dehydrogenase (LDH) release. *P < 0.05 vs. Ad-control. ^#^*P* < 0.05 vs. Ad-control + OGD, ANOVA; *n* = 4. **(E)** Apoptosis was detected using TUNEL assay. Quantitative analysis of TUNEL-positive cells from three independent experiments. *P < 0.05 vs. Ad-control. ^#^*P* < 0.05 vs. Ad-control + OGD, ANOVA; *n* = 4. **(F)** Neurons were infected and treated with OGD as in **(D)**. Western blots analysis of expression of cleaved PARP protein (upper panel). Quantitative analysis of cleaved PARP was shown in the bottom panel. *P < 0.05 vs. Ad-control. ^#^*P* < 0.05 vs. Ad-control + OGD, ANOVA; *n* = 4. **(G)** Western blots analysis of expression of Bax and Bcl-2 proteins (upper panel). Quantitative analysis of the ratio of Bax/Bcl-2 was shown in the bottom panel. *P < 0.05 vs. Ad-control. ^#^*P* < 0.05 vs. Ad-control + OGD, ANOVA; *n* = 4.

To investigate whether Nrdp1 is implicated in OGD-induced apoptosis in neurons, we transfected cerebral cortical neurons with Ad-control, Ad-Nrdp1 or Ad-si-Nrdp1. As shown in Figure 3, the transfection efficiency reached more than 90% at 24 h after transfection (Figure 3A), and western blot analysis showed that incubating the neurons with Ad-si-Nrdp1 and Ad-Nrdp1 for 48 h significantly reduced (~90% reduction) and increased (~2-fold increase) Nrdp1 protein levels, respectively (Figures 3B,C). We assessed the effect of Ad-si-Nrdp1 and Ad-Nrdp1 on OGD-induced neuronal death by measuring LDH release (indicating late apoptosis and necrosis) and TUNEL staining (indicating apoptosis). The data showed that cell death and apoptosis did not differ across the groups under basal conditions (Figures 3D,E and Supplementary Figure S2). However, after OGD treatment, inhibition of Nrdp1 significantly attenuated neuronal death and apoptosis as compared with the Ad-control, while transfection with the Ad-Nrdp1 greatly enhanced OGD-induced neurons death and apoptosis (Figures 3D,E and Supplementary Figure S2).

To further verify a role of Nrdp1 in OGD-induced apoptosis in neurons, we assessed the effect of Nrdp1 on several key apoptosis-associated signal proteins including cleaved-PARP and Bax/Bcl-2. As shown in Figures 3F,G, 6-h OGD induced a significant increase in cleaved PARP levels (PARP activation) and a greater ratio of Bax/Bcl-2 in cerebral cortical neurons, and transfection with Ad-si-Nrdp1 abolished these changes. Accordingly, overexpression of Nrdp1 augmented OGD-induced increases in cleaved PARP and Bax/Bcl-2 ratio. As expected, Ad-si-Nrdp1 or Ad-Nrdp1 alone did not affect these apoptosis-associated signal proteins (Figures 3F,G). Taken together, these results clearly indicate that Nrdp1 plays an important role in ischemia-induced apoptosis in cerebral cortical neurons.

### Effects of Nrdp1 on HIF-1α and USP8 Expression in Cerebral Cortical Neurons and PC12 Cells Exposed to OGD Treatment

**Figure 4:**
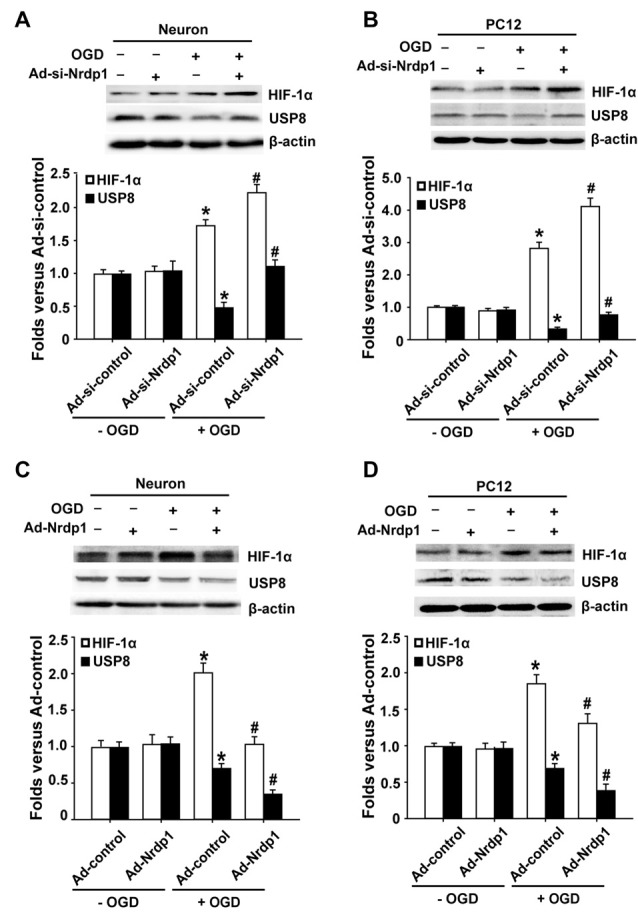
Effects of Nrdp1 expression on hypoxia inducible factor-1α (HIF-1α) and USP8 *in vitro* after OGD treatment. **(A,B)** Primary rat cerebral cortical neurons and PC12 cells were infected with Ad-si-control or Ad-si-Nrdp1 and exposed to 6-h OGD respectively. Western blot analysis of protein levels of HIF-1α and USP8 of ischemic neurons (top panels). Histograms show relative intensity of HIF-1α and USP8 (bottom panels). *P < 0.05 vs. Ad-si-control. ^#^*P* < 0.05 vs. Ad-si-control + OGD; *n* = 4. **(C,D)** Primary rat cerebral cortical neurons and PC12 cells were infected with Ad-control or Ad-Nrdp1 and exposed to 6-h OGD respectively. Western blot analysis of protein levels of HIF-1α and USP8 of ischemic neurons (top panels). Histograms show relative intensity of HIF-1α and USP8 (bottom panels). *P < 0.05 vs. Ad-control. ^#^*P* < 0.05 vs. Ad-control + OGD; *n* = 4.

HIF-1α acts as an intracellular sensor for hypoxia and promotes the cells to adapt to hypoxic/ischemic conditions (Zis et al., 2015), thus we hypothesized that Nrdp1 might interact with or suppress HIF-1α to promote neuronal cell death under OGD conditions. To test this possibility, we transfected primary cortical neurons and PC12 cells with Ad-control, Ad-Nrdp1, Ad-si-control or Ad-si-Nrdp1 before exposing to OGD for 6 h. HIF-1α protein levels were analyzed by western blot. As shown in Figures 4A,B, 6-h OGD induced a significant increase in the accumulation of HIF-1α protein in both primary neurons and PC12 cells transfected with Ad-si-control, and of note, this increase was further augmented or attenuated when Nrdp1 was knocked down by Ad-si-Nrdp1 or overexpressed by Ad-Nrdp1, respectively (Figures 4C,D). These data suggest that Nrdp1 may act as a negative regulator for HIF-1α expression in neurons under OGD conditions.

The fact that Nrdp1’s substrate USP8 can protect HIF-1α from pVHL-mediated degradation (Troilo et al., 2014) led us to hypothesize that Nrdp1 may act on USP8 to regulate HIF-1α expression under OGD condition. To test this, we investigated the change of USP8 in OGD-treated neurons and the impact of Ad-Nrdp1 and Ad-si-Nrdp1 on USP8 expression. As shown in Figures 4A,B, 6-h OGD induced a significant decrease in the level of USP8 proteins in the cells transfected with Ad-si-control. Importantly, this reduction was partially reversed when Nrdp1 was knocked down by Ad-si-Nrdp1, and was exacerbated when Nrdp1 was overexpressed by Ad-Nrdp1 (Figures 4C,D). Collectively, these results suggest that Nrdp1 may contribute to OGD-induced neuronal cell death via suppressing HIF-1α and USP8 expression.

### Nrdp1 Promotes Ubiquitin-Mediated Degradation of USP8 and Decreases its Interaction with HIF-1α

**Figure 5:**
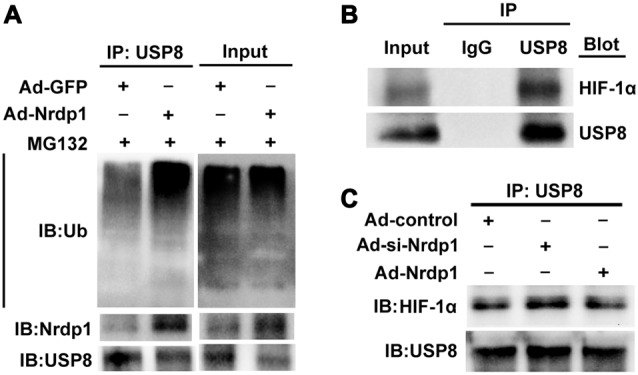
Nrdp1 accelerates ubiquitin-mediated degradation of USP8 and decreases its interaction with HIF-1α. **(A)** The lysates from Ad-GFP/Ad- Nrdp1 adenovirus PC12 cells were immune-precipitated with anti-USP8 antibody and analyzed by immunoblotted with anti-ubiquitin antibody to detect ubiquitylated forms of USP8 *in vitro*. **(B)** The interactions of USP8 with HIF-1α were detected with co-immunoprecipitation in PC12 cells under OGD treatment or **(C)** after transfected Ad-si-control/Ad-si-Nrdp1/Ad-Nrdp1 adenovirus. PC12 cells lysates were immune-precipitated with anti-USP8 antibody or control IgG, and the immune-precipitates were subjected to sodium dodecylsulfate-polyacrylamide gel electrophoresis and immunoblotted with anti-USP8 and anti-HIF-1α antibody.

Lastly, to ambiguously demonstrate the interactions between Nrdp1 and USP8 and between USP8 and HIF-1α in OGD-treated neurons, we performed co-immunoprecipitation experiments. Since Nrdp1 targets USP8 for ubiquitylation, we speculated that overexpression of Nrdp1 could enhance protein ubiquitylation and USP8 degradation in PC12 cells. To test this, we pulled down ubiquitylated species from PC12 cell extracts, and then detected the levels of protein ubiquitilytion in the presence of proteasome inhibitor MG132 as well as the protein levels of Nrdp1 and USP8. As shown in Figure 5A, overexpression of Nrdp1 by Ad-Nrdp1 significantly increased the whole levels of protein ubiquitylation in comparison to Ad-green fluorescent protein (GFP) control, and this change was accompanied by decreased USP8 protein levels.

To demonstrate a direct interaction between USP8 and HIF-1α in OGD-treated PC12 cells, we performed co-immunoprecipitation assays and found that HIF-1α was precipitated by antibody against USP8, but not by control rabbit IgG (Figure 5B). Moreover, under OGD conditions, overexpression of Nrdp1 by Ad-Nrdp1 reduced co-immunoprecipitation between USP8 and HIF-1α, while knockdown of Nrdp1 by Ad-si-Nrdp1 enhanced the interaction between these two proteins (Figure 5C). These data indicate that under ischemic conditions, Nrdp1 upregulation may hinder the stabilization of HIF-1α in neurons via promoting ubiquitin-mediated degradation of USP8, thus attenuating cellular adaptive response to hypoxia/ischemia.

## Discussion

The E3 ligase Nrdp1 has been extensively investigated on cell growth, apoptosis and inflammation in cancer cells and other cell types (Qiu et al., 2004; Wang et al., 2009; Ingalla et al., 2010; Byun et al., 2015). In the present study, we investigated Nrdp1’s role in ischemic neuronal injury. The major findings include: (1) Nrdp1 is significantly upregulated in the ischemic brain tissue and in OGD-treated neuronal cells; (2) overexpression or knockdown of Nrdp1 enhances or attenuates OGD-induced apoptosis in neurons, respectively, and these changes are accompanied by the downregulation or upregulation of Nrdp1’s substrate USP8; and (3) USP8 may directly interact with HIF-1α to prevent its degradation, and under OGD conditions, Nrdp1 may interfere with HIF-1α stabilization via promoting USP8 degradation. These data suggest that Nrdp1 may attenuate neuron’s adaptive response to hypoxia/ischemia via interfering USP8-mediated HIF-1α stabilization, thus contributing to neuronal death under ischemic conditions.

Apoptotic neuronal death is a common event accounting for neuron loss in ischemic stroke (Cao et al., 2004; Widiapradja et al., 2012; Wang et al., 2014). Therefore, the mechanism of neuronal apoptosis under ischemic conditions has been an important research focus in the past decades. Deregulation of the UPS is believed to be an important contributor to ischemic neuronal injury (Wojcik and Di Napoli, 2004; Doeppner et al., 2016). The E3 ligase Nrdp1 has been recently shown to mediate neuronal apoptosis through reducing BRUCE expression in LPS-induced neuroinflammation (Shen et al., 2015). Our previous study has also demonstrated a role of Nrdp1 in promoting cardiac myocyte apoptosis in experimental I/R (Zhang et al., 2011b). Here our *in vivo* and *in vitro* data show that ischemia induces Nrdp1 upregulation in cerebral cortical neurons. Of note, this change is quite rapid and persistent, as Nrdp1 mRNA expression is increased in neurons at 1 h after OGD treatment and remains high at the end of 6-h OGD exposure. Our data that knockdown of Nrdp1 with siRNA reduces OGD-induced cell death/apoptosis and overexpression of Nrdp1 by Ad-Nrdp1 increases neuronal death clearly supports a role of Nrdp1 in ischemic neuronal injury. Moreover, along with Nrdp1 knockdown or overexpression is the inhibition or activation of apoptosis-associated proteins, including caspase-3, PARP-1, Bax/Bcl-2 ratio, further supporting a proapoptotic action of Nrdp1 in OGD-induced neuron injury.

Nrdp1 is inducible in cells in response to different stimuli, and its stability largely relies on its substrate USP8, a de-ubiquitinating enzyme (Wu et al., 2004). Thus, Nrdp1 and USP8 may restrict each other (De Ceuninck et al., 2013). When Nrdp1 is increased, more USP8 will be degraded by Nrdp1, and as a return, less USP8 will make Nrdp1 unstable, resulting in less Nrdp1 and more USP8 in the cells. Consistently, here our data also show that under OGD condition, Nrdp1 upregulation concurrently occurs with USB8 downregulation in neuronal cells. Moreover, Nrdp1 overexpression augments OGD-induced USP8 downregulation, while knockdown of Nrdp1 ameliorates this effect. Although we did not design experiments to verify USP8’s effect in stabilizing Nrdp1 protein in neurons under ischemic conditions, our data clearly demonstrate that the interaction between these two proteins is associated with OGD-induced neuronal death.

Mounting evidence suggests that HIF-1α is an essential transcriptional regulator of various vital processes in neurons including the adaptation of cells to hypoxic environments (Barteczek et al., 2017), cell proliferation (Zhang et al., 2017), cell apoptosis (Yin J. et al., 2017) and metabolism (Carmeliet et al., 1998; Cho et al., 2015). In the brain, HIF-1α has been reported to act as a pivotal protective regulator in ischemic brain injury (Fan et al., 2009; Singh et al., 2012; Zhang et al., 2014). Baranova et al. (2007) found that knockdown of neuronal HIF-1α enhances ischemic brain injury. Activation of HIF-1α-associated signaling cascades, such as EPO pathway (Liu et al., 2006; Ryou et al., 2012) and VEGF pathway (Yin W. et al., 2017) in neurons could protect the brain from I/R damage through increasing microvascular density and/or restoring local blood flow and oxygen supply. Yang X. S. et al. (2017) found that HIF-1α involved in necroptosis contributed to ischemic brain injury induced by OGD and MCAO. Inhibition of the 20S proteasomal activity can protects HIF-1α from degradation and provides neuroprotection in cerebral ischemia (Badawi and Shi, 2017). Here we show that 6-h OGD without re-oxygenation induces HIF-1α protein accumulation in neuronal cells, and this change is enhanced or suppressed by overexpression or knockdown of Nrdp1, respectively. This important observation has evoked us to further explore the interaction between Nrdp1 and HIF-1α in OGD-treated neurons.

USP8 can protect HIF-1α from degradation mediated by E3 ubiquitin ligase pVHL via de-ubiquitination (Troilo et al., 2014), which promoted us to hypothesize that USP8 may be an important bridge molecule that mediates the interaction between Nrdp1 and HIF-1α. Indeed, we observed two simultaneous changes in OGD-treated neurons that support our hypothesis. First, Nrdp1 overexpression leads to increased protein ubiquitylation and suppressed interaction between Nrdp1 and USP8 (due to increased USP8 degradation). Second, USP8 directly interacts with HIF-1α, and this interaction is increased when Nrdp1 is knocked down. The interaction between USP8 and HIF-1α has been previously reported by Troilo et al. (2014). Our data suggest that under ischemic conditions, Nrdp1 upregulation may lead to an accelerated degradation of USP8, which in turn attenuates USP8’s capability to protect HIF-1α against pVHL-mediated degradation, thus interfering neuronal cells to timely adapt to hypoxic/ischemic conditions. In addition, our data also suggest that HIF-1α is an important downstream effector molecule in the pathway of Nrdp1-mediated apoptosis during ischemic neuronal injury. Future studies are warranted to explore the mechanisms underlying enhanced Nrdp1 expression under ischemic conditions.

It is worth pointing out one important fact, that is, whether HIF-1α is protective or detrimental in ischemic stroke depends on the stroke stage, ischemia severity and ischemia duration (Yang et al., 2013). For example, there are studies showing that HIF-1α knockdown protects the brain against ischemic damage (Helton et al., 2005). However, other studies have reported that inhibition of HIF-1α and HIF-2α is beneficial to the neurons in the very acute phase after ischemic stroke (Barteczek et al., 2017). Under our experimental conditions, HIF-1α may be more likely a good molecule in promoting neuronal cells to rapidly adapt hypoxic conditions. However, future experiments are needed to demonstrate this speculation.

Taken together, the present study demonstrates that in response to ischemic stimuli, Nrdp1 is upregulated in neurons and contributes to ischemic neuronal death, and this effect may be associated with suppressed adaptive response to hypoxia/ischemia due to accelerated USP8 degradation and HIF-1α destabilization. Therapeutic strategies that target Nrdp1 activation may provide neuroprotection against ischemic brain injury.

## Author Contributions

This work was performed and accomplished by all authors. YZ, KY and TW contributed to the execution of the entire research project, WLi performed the statistical analysis. YZ, XJ and WLiu wrote the manuscript. All authors approved the final manuscript.

## Conflict of Interest Statement

The authors declare that the research was conducted in the absence of any commercial or financial relationships that could be construed as a potential conflict of interest.

## Supplementary Material

FIGURE S1 Nerve growth factor (NGF)-induced differentiation of PC12 cells. Immunofluorescence staining for microtubule associated protein 2 (MAP-2) antibody (red) was performed to induce PC12 cells differentiation after the 7 days supplement of NGF (magnification, ×400).

FIGURE S2 Effects of neuregulin receptor degradation protein-1 (Nrdp1) on oxygen-glucose deprivation (OGD)-induced apoptosis in primary rat cerebral cortical neurons by TUNEL assay. TUNEL assay showed that 6 h OGD significantly increased the number of TUNEL-positive apoptotic neuron nuclei and knockdown of Nrdp1 significantly decreased this increase, but overexpression of Nrdp1 enhanced this increase. Representative micrographs of Phase contrast, DAPI (blue, counter staining) and TUNEL (red fluorescence) (magnification, ×200).

## Abbreviations

BBB: blood brain barrier
DMEM: dulbecco’s modified eagle medium
FBS: fetal bovine serum
GFP: green fluorescent protein
HIF-1α: hypoxia inducible factor-1α
LDH: lactate dehydrogenase
LPS: lipopolysaccharide
MCAO: middle cerebral artery occlusion
NGF: nerve growth factor
OGD: oxygen-glucose deprivation
PLL: poly-l-lysine
TTC: 2,3,5-triphenyltetrazolium chloride
UPS: ubiquitin-proteasome system
USP8: ubiquitin-specific protease 8.
