# Supplementary figures and images for: Nrdp1 Increases Ischemia Induced Primary Rat Cerebral Cortical Neurons and Pheochromocytoma Cells Apoptosis Via Downregulation of HIF-1α Protein
Source: Front Cell Neurosci. 2017 Sep 20;11:293. doi: 10.3389/fncel.2017.00293 (PMC5611384; doi:10.3389/fncel.2017.00293)

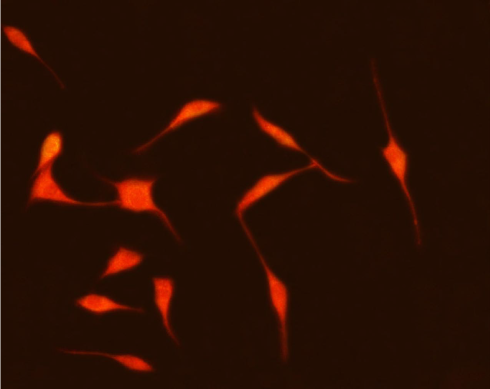

Supplement: FIGURE S1 — Nerve growth factor (NGF)-induced differentiation of PC12 cells. Immunofluorescence staining for microtubule associated protein 2 (MAP-2) antibody (red) was performed to induce PC12 cells differentiation after the 7 days supplement of NGF (magnification, ×400). [file Image_1.tif]

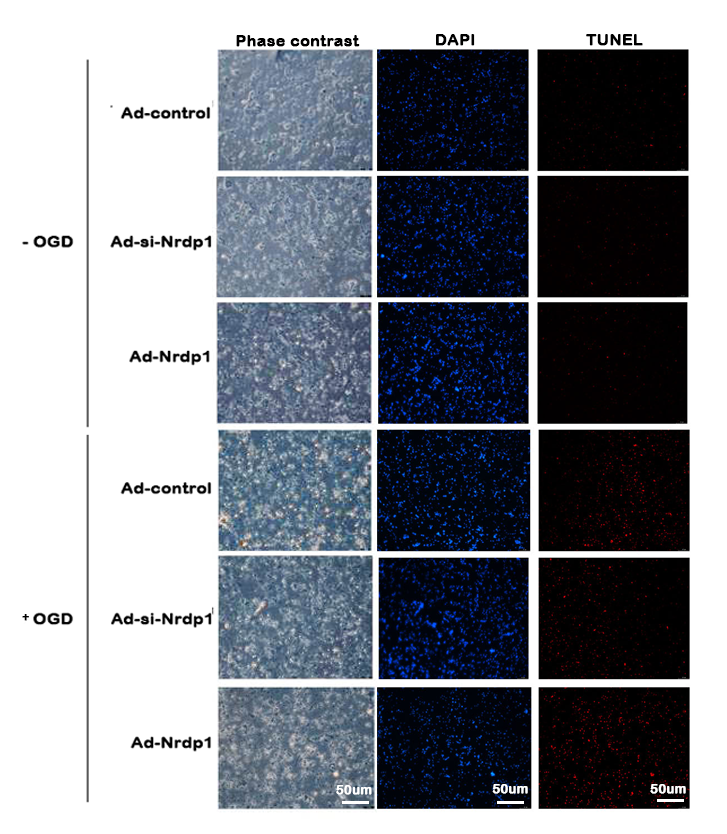

Supplement: FIGURE S2 — Effects of neuregulin receptor degradation protein-1 (Nrdp1) on oxygen-glucose deprivation (OGD)-induced apoptosis in primary rat cerebral cortical neurons by TUNEL assay. TUNEL assay showed that 6 h OGD significantly increased the number of TUNEL-positive apoptotic neuron nuclei and knockdown of Nrdp1 significantly decreased this increase, but overexpression of Nrdp1 enhanced this increase. Representative micrographs of Phase contrast, DAPI (blue, counter staining) and TUNEL (red fluorescence) (magnification, ×200). [file Image_2.tif]
